# Monitoring the Prevalence of Antimicrobial Resistance in Companion Animals: Results from Clinical Isolates in an Italian University Veterinary Hospital

**DOI:** 10.1155/2023/6695493

**Published:** 2023-09-14

**Authors:** Raffaele Scarpellini, Giammarco Assirelli, Massimo Giunti, Erika Esposito, Elisabetta Mondo, Silvia Piva

**Affiliations:** Department of Veterinary Medical Sciences, University of Bologna, Via Tolara di Sopra 50, Bologna 40064, Italy

## Abstract

The role of companion animals in the spread of antimicrobial resistance (AMR) is still not well known. As part of a wider surveillance system, this study aimed to provide data about AMR in bacterial isolates from infections in companion animals referred to an Italian University Veterinary Hospital (VUH) from November 2020 to September 2022. A total of 940 isolates were identified with MALDI-TOF MS and subsequently the antimicrobial susceptibility test (AST) for 12 antimicrobials was performed. Urine was the most commonly submitted specimen (54.92%) and *Escherichia coli* was the most common bacterial species isolated (36.06%). Out of the 940 isolates, 747 (79.47%) were nonsusceptible to at least one drug (AMR), while 420 (44.68%) were considered multidrug resistant (MDR). The highest nonsusceptibility percentages were recorded for clindamycin (59.65 %), erythromycin (58.96 %), ampicillin (52.85%), and enrofloxacin (48.19%). Alarming percentages were recorded also for ceftiofur (25.51%), amoxicillin–clavulanate (22.99%), and piperacillin–tazobactam (15.85%). In multivariable risk factors analysis, previous use of invasive devices (*p* < 0.01 in both cases) and previous use of antimicrobials (*p* < 0.01 in both cases) were statistically related with higher AMR and MDR percentages. Apart from a general evaluation, the study focused on specific bacterial species (*Enterococcus faecium*, *Staphylococcus aureus*, *Klebsiella pneumoniae*, *Acinetobacter baumannii*, *Pseudomonas aeruginosa*, and *Enterobacter* spp.) and specimens (blood cultures, urine from suspected healthcare-associated urinary tract infections, and surgical site infections), showing in both cases higher AMR and MDR percentages compared to average. These data highlight the urgency to further investigate AMR spread in pets and how passive surveillance systems can be effective tools to monitor AMR and to optimize antimicrobial use.

## 1. Introduction

Bacterial antimicrobial resistance (AMR) is considered one of the most concerning and widespread health concerns worldwide. In a global epidemiological overview, limited data are available regarding the role of companion animals in the spread of AMR bacteria [1]. This topic is particularly relevant in European Union countries, in which pets are increasingly popular with more than 60 million cats and dogs [2] sharing the same environment with people. This must be considered not only for the increasing risk of therapeutic failure in animals, but also for the wide use of antimicrobials shared with human medicine and the potential zoonotic transmission of AMR [3]. In this context, veterinary hospitals could play a major role in the selection of multidrug resistant (MDR) bacteria, which could spread into the local community not only through animals, but also owners or veterinary personnel. From a one health perspective, some bacterial species (*Enterococcus faecium*, *Staphylococcus aureus*, *Klebsiella pneumoniae*, *Acinetobacter baumannii*, *Pseudomonas aeruginosa*, and *Enterobacter* spp.) grouped under the acronym ESKAPE [4] represent a particular matter of concern due to their pathogenic potential. In addition, MDR bacteria represent a threat because they are often associated with severe infections such as bloodstream infections, surgical site infections (SSIs), or healthcare-associated urinary tract infections (HA UTIs). In Europe, some countries are developing national surveillance programs in small animal practice [5]; however, there is still a lack of specific legislation.

For all these reasons, the need to focus on antimicrobial stewardship measures, guidelines, and education should be considered with urgency in small animal practice. The first step is data collection, which can be executed through internal passive surveillance systems by registering information from clinical samples for diagnostic purposes, with a favorable cost-effectiveness ratio and less time-consuming efforts [6]. The aims of this paper are: (i) to describe a passive surveillance system for AMR in bacterial isolates from companion animals referred to a Veterinary University Hospital (VUH), (ii) to describe the specimen type and species distribution of the analyzed isolates, their AMR and MDR percentages and their nonsusceptibility percentages in relation with the 12 antimicrobials tested; (iii) to identify the risk factors associated with AMR/MDR in the analyzed isolates; (iv) to focus on the nonsusceptibility percentages of isolates identified as species from the ESKAPE group, and of isolates from specimens associated with severe infections such as bloodstream infections, suspected HA UTIs, and SSIs.

## 2. Materials and Methods

### 2.1. Study Design

This prospective, observational study was part of a larger project on surveillance of AMR and hospital-acquired infections in VUHs. The data used for the analysis originated from routine diagnostic bacteriological cases received by the Veterinary Laboratory of Bacteriology (VeLaBac) at the Department of Veterinary Medical Sciences, from the internal Small Animals VUH. We collected data from November 2020 to September 2022 on isolates from biological specimens from dogs and cats referred to the VUH, submitted for culture, bacterial isolation, identification, and antimicrobial susceptibility testing (AST). Only isolates with a reported AST result were included in the study.

### 2.2. Culture and Identification of Bacterial Isolates

Depending on the specimen type, standard microbiological procedures were used and are listed in Table S1. The different specimen types, sampled by the submitting veterinarian, were classified as follows: abdominal/pleural/peritoneal effusion, abscess, bile, biopsy, blood culture, ear swab, exudate, nasal swab, respiratory tract lavage, urine (both by cystocentesis and from catheterization), SSI and implant, wound, vaginal/uterine swab, and “other.” The “other” cathegory included specimens with very few frequencies or without specific indications. After the incubation of 24–48 hr at 37 + −1°C, plates that presented adequate growth according to guidelines [7] were considered positive. Colonies were evaluated morphologically and the identification at species level of each isolate was assessed using the matrix-assisted laser desorption–ionization time-of-flight mass spectrometry method (MALDI-TOF MS) (Biotyper, Bruker Daltonics, Billerica, MA), following manufacturer's instructions (Bruker Daltonik, Bremen, Germany) and considered a species-level identification when the ID score was >2 (green—high accuracy). If isolates identified as the same bacterial species were isolated from different specimens in the same patient at the same time, they were considered separately only in the specimen analysis.

### 2.3. Data Collection

For every isolate, the following variables were collected from the patient: species (dog/cat), age (years, rounded up), type of specimen (see above), previous hospitalization/surgery in the past 30 days (yes/no), hospitalization at the time of the sampling (yes/no), hospitalization in intensive care unit (yes/no), surgery at the time of the sampling (yes/no), days of hospitalization at the time of the sampling (number of days), invasive device use in the previous 90 days (yes/no), antimicrobial use in the past 90 days (yes/no), and current antimicrobial use (yes/no).

### 2.4. Antimicrobial Susceptibility Testing

AST of all the isolates was performed using the Kirby–Bauer disc diffusion method, according to the Clinical and Laboratory Standard Institute (CLSI) guidelines [8]. For every tested drug, each isolate was classified as susceptible (S), intermediate (I), or resistant (R) based on the CLSI [8] veterinary breakpoints. Overall, 12 antimicrobials from 8 antimicrobial classes were included in the final analysis (Table S2). All the discs were purchased from Oxoid (Oxoid, Milan, Italy). For Gram-negative bacteria, clindamycin and erythromycin were not tested due to their low-activity rates [9]. For certain species, antimicrobial agents known to exhibit intrinsic resistance phenotypes according to the National Reference Laboratory for AMR [10] were not tested. Isolates identified as the same bacterial species found in the same patient at different time points were considered separately only when they showed different AST results. For AST interpretation, the strains were divided into “susceptible” and “nonsusceptible,” as suggested by Sweeney et al. [11], where the “nonsusceptible” category included resistant and intermediate isolates. Isolates that were nonsusceptible to at least one antimicrobial drug were considered as AMR isolates, and isolates that were not susceptible to at least one antimicrobial drug in three or more antimicrobial classes were considered as MDR [12].

### 2.5. Statistical Analysis

Descriptive statistics was performed considering bacterial species and their Gram stain, type of specimen, nonsusceptibility percentages of the 12 tested drugs, and AMR and MDR proportions, that were evaluated by dividing the number of AMR/MDR strains by the number of total strains. Besides this general overview, descriptive statistics was also prepared considering only bacterial species belonging to the ESKAPE group and for strains from specific specimens such as blood cultures, urine from suspected HA UTIs, following the definition by Haque et al. [13], SSIs/surgical implants. The association between Gram stain and AMR/MDR was tested with the *χ*^2^ test. The alpha risk was set to 0.05. The association between AMR/MDR and the independent variables was calculated using univariable logistic regression analyses. Categorical variables (species, previous hospitalization/surgery, hospitalization at the moment of the sampling, intensive care unit hospitalization, surgery at the time of the sampling, previous invasive device use, previous antimicrobial treatment, and antimicrobial treatment at the time of the sampling) were expressed as percentages. Normality and heteroskedasticity of data were assessed with the Shapiro–Wilk test and the Levene's test. Only variables with a *p* < 0.05 in the initial assessment were included in the multivariable logistic model built using forward stepwise selection at *p* < 0.05. Data were checked for multicollinearity with the Belsley–Kuh–Welsch technique. Odds ratios (OR) and 95% confidence intervals (CI) were calculated. Statistical analysis was performed with MedCalc (version v22.009).

## 3. Results

From a total of 2,406 specimens from 1,759 patients (1,296 dogs and 463 cats), 863 (35.87%, from 673 patients, 596 dogs and 167 cats) were positive for bacterial growth and a total of 940 strains with a reported AST was recorded. The specimen type distribution is listed in Table 1. Urine (*n* = 474, 54.92%) was the most frequent specimen in both cats and dogs, followed by ear swab in dogs (*n* = 69, 10.00%) and blood culture (*n* = 14, 8.09%) in cats.

Strains isolated from cats were 20.64% (*n* = 194) of the total, while 79.36% (*n* = 746) were isolated from dogs. Distribution of bacterial species is shown in Table 2. Five hundred eighty-four (62.13%) strains were classified as Gram negative, while 356 (37.87%) as Gram positive. *Escherichia coli* (36.06%) was the most commonly isolated species, followed by *Staphylococcus pseudintermedius* (12.87%), *Streptococcus canis* (7.23%), and *Enterococcus faecalis* (5.11%). *S. pseudintermedius* was less frequent in cats than in dogs (5.15% vs. 14.88%), while *E. faecalis* was more frequent in cats (9.28% vs. 4.02%).

The overall AMR percentage was 79.47% (*n* = 747), while MDR percentage was 44.68% (*n* = 420). Considering only AMR strains, 56.22% had an MDR pattern. The AMR percentages were, respectively, 73.12% and 89.89% in Gram-negative and Gram-positive isolates (OR = 3.27; CI 95% 2.21–4.83; *p* < 0.01), while the MDR percentages were, respectively, 35.79% and 59.27% (OR = 2.61; CI 95% 1.99–3.42; *p* < 0.01). The single resistance analysis for each drug tested is shown in Table 3. The highest nonsusceptibility percentages were recorded for clindamycin, erythromycin, ampicillin, and enrofloxacin (59.65%, 58.96%, 52.85%, and 48.19%, respectively). The lowest nonsusceptibility percentages were recorded for amikacin and piperacillin–tazobactam (11.32% and 15.85%, respectively), and none of the tested drug had nonsusceptibility percentages lower than 10%.

Univariable analysis results for AMR and MDR are shown in Table 4. In the multivariable analysis for AMR (Table 5), species dog (OR 1.55, CI 95% 1.06–2.29; *p*=0.02), invasive device use in the previous 90 days (OR 2.06, CI 95% 1.29–3.30, *p* < 0.01), and antimicrobial use in the past 90 days (OR 3.74, CI 95% 2.52–5.56, *p* < 0.01) were associated with a higher proportion of AMR. In the multivariable analysis for MDR (Table 5), invasive device use in the previous 90 days (OR 1.76, CI 95% 1.28–2.41, *p* < 0.01), antimicrobial use in the past 90 days (OR 2.47, 95% CI 1.93–3.15, *p* < 0.01), and current antimicrobial use (OR 1.39, 95% CI 1.08–1.79, *p*=0.01) were associated with higher percentages of MDR.

Considering only the identified strains belonging to the ESKAPE group (*n* = 169, 17.98% of the total strains), AMR and MDR percentages were 88.17% (149) and 49.70% (84), respectively. The single percentage of nonsusceptibility is presented in Table 6. *Klebsiella pneumoniae* was the most commonly isolated (*n* = 47), followed by *P. aeruginosa* (*n* = 45) and *Enterobacter* spp. (*n* = 42). *P. aeruginosa*, *A. baumanni*, and *E. faecium* strains showed the highest AMR (100.00%) percentages, while *E. faecium* showed the highest MDR percentage (90.00%), followed by *K. pneumoniae* (57.45%).

Considering only strains isolated from blood cultures, urines from suspected HA UTIs and SSIs/surgical implants, they represented 14.68% (*n* = 138) of the total strains. Strains from blood cultures and SSIs/surgical implants (*n* = 49) accounted for 5.21%, while urines from suspected HA UTIs (*n* = 42) accounted for 4.47%. In two cases, the same strain was isolated from blood culture and urine from suspected HA UTI in the same patient. AMR and MDR percentages were considerably higher than the average ones observed in all the isolates (Table 7). Considering single analysis for each tested drug, the nonsusceptibility percentage toward ceftiofur was the one with the largest difference compared with the average percentage in both blood cultures (46.67% vs. 25.51%) and SSIs and surgical implants (59.52% vs. 25.51%), while it was cefazolin/cephalothin in urines from suspected HAIs (61.29% vs. 30.73%). Considering drugs with the lowest nonsusceptibility percentages between the 12 tested, a considerable difference was observed for piperacillin–tazobactam, with nonsusceptibility percentages from 24.49% (blood cultures) to 34.69% (SSIs/surgical implants), compared with the average nonsusceptibility percentage for piperacillin–tazobactam of 15.85%.

## 4. Discussion

This study describes the most frequently isolated bacteria from cat and dog infections sampled from November 2020 to September 2022 in an Italian VUH, their AMR/MDR percentages and associated risk factors, focusing on the specific bacterial species (ESKAPE group) and specimens, through an internal passive surveillance system.

In both cats and dogs, the most frequently submitted sample was urine (54.92% of the total). Although this result is in contrast with other similar studies worldwide in which it was found to be wounds [14–16], or ear swabs in dogs [17], its high prevalence was expected in Italy because it is considered to be one of the most frequently cultured biological materials [18]. Ear swabs (8.93% of total) were more frequent in dogs compared with cats (10.00% vs. 4.62%), while in contrast nasal swabs were more frequent in cats than in dogs (6.94% vs. 1.30%). Those results are comparable with a Spanish study [16] over 5,875 clinical samples from dogs and cats, in which otitis was shown to be more prevalent in dogs and respiratory tract disease in cats. Considering bacterial species analysis, *E. coli* was found to be the most common in both cats and dogs. Its high prevalence is probably related with the high prevalence of urine specimens. Indeed, other studies worldwide have highlighted that *E. coli* is the most common agent in urinary tract infections in pets [19–22]. *S. pseudintermedius*, found to be the second most common species in dog infections, it has been reported to be a common agent of otitis and pyoderma in small animal practice [23]; notably, its prevalence is higher than *S. aureus*, as highlighted by the other works [24–27].

Dogs and cats were considered together for the general evaluation of AMR and MDR percentages. In contrast with other studies [17, 28], a temporal evaluation of resistance trends was not performed because of the short timeframe of the study, but it could be implemented in future works. Since the majority of the studies on AMR in companion animals have been conducted on specific bacterial species or specimens, the originality of this study was to consider all the isolates from a determined timeframe. A recent Colombian study over a 4-year period on 1,413 isolates from dogs and cats showed MDR percentages of 18.7% and 22%, respectively [14], whereas a Malaysian study on 780 isolates from 2015 to 2017 reported MDR percentages of 75.8% in cats and 71.6% in dogs [15]. A longer Canadian study from 1994 to 2013 on more than 15,000 specimens showed MDR rates of 9% and 12% in cats and dogs isolates, respectively [17]. In Italy, Smoglica et al. [29] described very similar results to our report in isolates from urines in companion animals, with AMR and MDR rates of 74.3% and 44.8%, respectively. Since the reasons that could explain the difference between the results are primarily geographical, comparisons, and data extrapolation should be done with prudence. The high nonsusceptibility percentages have to be considered as a reflection of the Italian epidemiological situation about AMR, which is currently one of the most concerning in Europe [30]. AMR and MDR occurrence in companion animals is influenced by policies in antimicrobial use in the local community, not only in small animal settings but also in livestock production and human medicine [31]. Notably, the present study was conducted in a VUH, which is considered a higher risk place due to the large number of personnel and students [32], and high number of referred patients, that are more likely to have received previous antimicrobic treatment [33, 34]. This could mean that the percentages reported in this study could be higher if compared with the other veterinary settings from the same geographical area. Moreover, the study describes only bacterial strains from diagnostic cases, so it does not provide an overall picture about AMR in the general dogs and cats population.

Considering the nonsusceptibility for the single drug, this study highlights that clindamycin, amoxicillin–clavulanate, ceftiofur, piperacillin–tazobactam, and enrofloxacin percentages represent a serious threat from a one health perspective. In Europe, reported clindamycin nonsusceptibility percentages in pets reached a maximum of 100% in 39 *S. aureus* isolates from canine dermatitis in an Italian study [35]. Amoxicillin–clavulanate nonsusceptibility percentages, reported to be 22.99% in our study, are comparable the ones reported both by the French and the German national surveillance systems for AMR in companion animals, with percentages of nonsusceptibility of 35.5%–40% for *E. coli* in France [36], and of 20% in *E. coli* isolated from dogs with UTIs in Germany [37]. Notably, the nonsusceptibility percentage for ceftiofur (25.51%) are very similar to the percentage of *E. coli* resistant to third generation cephalosporins in the Emilia–Romagna region (22.6%) reported by the Italian surveillance system for AMR in humans [38]. Reports on piperacillin–tazobactam rates are scarcely present in veterinary literature, but the report from the Italian surveillance system in humans described nonsusceptibility percentages from 8.2% for *E. coli* to 44.8% for *K. pneumoniae* [38]. Considering enrofloxacin, the high nonsusceptibility percentages are not surprising since fluoroquinolones are one of the most commonly prescribed antimicrobial classes in small animal practice [39]. In Italy, a study on 70 *S. pseudintermedius* isolates from canine pyoderma [40] described a resistance rate of 94%.

Overall, these results reflect the widespread consumption of antimicrobials such as clindamycin, amoxicillin–clavulanate, ceftiofur, piperacillin–tazobactam, and enrofloxacin in the Italian small animal practice. Although the major limitation of the study is the absence of data on single drug prescriptions and consumption, from our results it can be assumed that nonsusceptibility percentages are proportional to them. In addition, risk factor analysis for AMR and MDR confirms the extremely strict relationship between antimicrobial use and the onset of resistance, as reported in other studies in both human [41] and veterinary medicine [42, 43]. Also, the use of invasive devices, related with the occurrence of HAIs in other studies [44], was linked with a higher chance of AMR and MDR strains. Compared to dogs, cats seems to have a lower predisposition to develop an infection caused by AMR bacteria; this result could be due to the differences in population size between the two species, but it may also reflect less frequent use of antimicrobials in cats, as described by Hur et al. [45] in Australia and by Joosten [46] in Italy in a multicentric study. In contrast, other studies by Escher et al. [47] and Singleton et al. [48], described a higher systemic antimicrobial prescription and AMR rates in cats than in dogs.

Isolated strains belonging to the ESKAPE group (*n* = 169) represented 17.98% of the total strains. AMR and MDR percentages were alarmingly high in every species considered. Particular attention should be paid to the species that were isolated more frequently, such as *K. pneumoniae*, *P. aeruginosa*, and *Enterobacter* spp., which accounted for 14.25% of the total strains. The isolates of *K. pneumoniae* had high nonsusceptibility percentages toward potentiated penicillins, very similar to the ones described by the Italian national surveillance system in humans [38], with percentages of 54.2% for amoxicillin–clavulanate and 44.8% for piperacillin–tazobactam. The high nonsusceptibility percentage toward enrofloxacin described in this study (70.21%) should also be considered, since it is very similar to a Portuguese study by Marques et al. [49], that reported resistance rates of 72% for enrofloxacin and ciprofloxacin in 25 *K. pneumoniae* isolates from pets with UTI. Notably, *P. aeruginosa* strains showed a lower MDR percentage, mainly due to the high number of intrinsic resistances (7 out of 12) toward the antimicrobials considered. The relatively low nonsusceptibility percentages toward amikacin and gentamicin, similar to the ones reported by the Italian national surveillance system in humans (3.6%–11.6%) [38] suggests that aminoglycosides should be considered when facing a pseudomonal infection, as confirmed by previous studies [16, 17]. For *Enterobacter* spp. isolates, their importance must be considered in relation to their high nonsusceptibility proportion even in the presence of several intrinsic resistances (5 out of 12). In particular, nonsusceptibility percentages toward third-generation cephalosporins such as ceftiofur (47.62%) recorded for *Enterobacter* spp. are higher than the results from a French study by Haenni et al. [50], in which it ranged from 3.5% to 10.2%. Considering *E. faecium* strains, the high nonsusceptibility percentages for penicillins +/− beta-lactamases inhibitors (83.33%–77.78%) are comparable with the ones for ampicillin (89.7%) described by the Italian surveillance system in humans [38] and suggest that, in contrast to other studies [17], the empiric treatment with this antimicrobial class in case of enterococcal infections should be avoided. Data about the ESKAPE group should address veterinary hospitals and clinics to enforce surveillance for these specific pathogens.

Considering only strains from bloodstream infections, suspected HA UTIs and SSIs, they represented 14.68% of the total. The higher AMR and MDR percentages compared with the average were expected, with relevant increases registered for piperacillin–tazobactam and cephalosporins. These results should remark the importance of a correct antimicrobial stewardship policy to avoid therapeutic failure in severe infections, in which antimicrobial use is required to save the patient's life.

## 5. Conclusions

In conclusion, the present article aimed to highlight that the role of companion animals in AMR epidemiology requires more attention, especially because the antimicrobials used are shared with human medicine. Considering the absence of collective control programs in the EU for companion animals, facilities such as veterinary hospitals should be encouraged to develop internal microbiological surveillance plans to obtain and to share data. Tailored surveillance plans can be useful to detect specific AMR frameworks and to serve as a guide for clinicians to make rational therapeutic decisions and to limit the selection and spread of AMR in the local community. Surveillance may also focus only on specific specimens or also bacterial species such as the ESKAPE group, which are extremely important from one health perspective, in order to prevent and address potential emerging health threats at the human–animal–environment interface.

## Figures and Tables

**Table 1:** Distribution of the 863 specimens (total number and percentage) considering specimen type, from November 2020 to September 2022.

Specimen type	Total number	(%)	Dogs (690)	(%)	Cats (173)	(%)	Percentage of positive specimens (%)
Urine	474	54.92	383	55.51	91	52.60	32.38
Abdominal/pleural/peritoneal effusion	26	3.01	19	2.75	7	4.05	39.39
Bile	9	1.04	6	0.87	3	1.73	13.04
Biopsy	24	2.78	21	3.04	3	1.73	33.33
Blood culture	49	5.68	35	5.07	14	8.09	16.39
Ear swab	77	8.93	69	10.00	8	4.62	61.11
Exudate	15	1.74	10	1.45	5	2.89	53.57
Nasal swab	21	2.43	9	1.30	12	6.94	70.00
Respiratory tract lavage	7	0.81	7	1.01	0	0.00	31.82
Surgical site infection (SSI) and implants	36	4.17	31	4.49	5	2.89	67.92
Vaginal/uterine swab	43	4.98	34	4.93	9	5.20	76.79
Wound	41	4.75	29	4.20	12	6.94	78.85
Abcess	13	1.51	11	1.59	2	1.16	59.09
Other	28	3.24	26	3.77	2	1.16	59.57

*Note*: distribution of specimen type in dogs (*n* = 690) and cats (*n* = 173) is reported, and also the percentage of submitted specimens that tested positive.

**Table 2:** Distribution of the 940 analyzed isolates based on bacterial species identification, from November 2020 to September 2022.

	Total isolates	(%)	dogs (746)	(%)	Cats (194)	(%)	(%) AMR (n.)	(%) MDR (n.)
*E. cloacae*	37	3.93	30	4.02	7	3.61	70.27 (26)	40.54% (15)
*E. faecalis*	48	5.11	30	4.02	18	9.28	95.83 (46)	27.08% (13)
*E. faecium*	18	1.91	14	1.88	4	2.06	100 (18/18)	88.89% (16/18)
*E. coli*	339	36.06	278	37.26	61	31.44	75.81% (257)	37.76% (128)
*K. pneumoniae*	47	5.00	40	5.36	7	3.61	85.11% (40/47)	57.45% (27/47)
others	116	12.34	82	10.99	34	17.53	75.86% (88)	39.66% (46)
*Pasteurella multocida*	12	1.28	10	1.34	2	1.03	16.67% (2)	8.33% (1)
*Proteus mirabilis*	46	4.89	35	4.69	11	5.67	50.00% (23)	21.74% (10)
*P. aeruginosa*	45	4.79	36	4.83	9	4.64	100% (45/45)	28.89% (13/45)
*S. aureus*	15	1.60	9	1.21	6	3.09	93.33% (14/15)	53.33% (8/15)
*Staphylococcus felis*	12	1.28	0	0	12	6.18	50.00% (6)	16.67% (2/12)
*Staphylococcus intermedius*	16	1.70	14	1.88	2	1.03	81.25% (13)	56.25% (9)
*Staphylococcus pseudintermedius*	121	12.87	111	14.88	10	5.15	89.26% (108)	74.38% (90)
*Streptococcus canis*	68	7.23	57	7.64	11	5.67	89.71 (61)	61.76% (42)

*Note*: the total number of isolates and the percentage are reported. Species distribution considering dogs (*n* = 746) and cats (*n* = 194) is also described. For every bacterial species considered, the percentage and the total number of AMR and MDR isolates is reported. The row “others” includes bacterial species with less than 10 isolates.

**Table 3:** Nonsusceptibility percentages for every single tested antimicrobial of the 940 analyzed isolates, from November 2020 to September 2022.

	No. of isolates tested (% of total isolates)	Total % of nonsusceptible isolates (total number of nonsusceptible isolates)	Gram + (%)	Gram− (%)
Amikacin	795 (84.57)	11.32 (90)	6.63% (14/211)	13.01% (76/584)
Gentamicin	863 (91.81)	23.06 (199)	33.33% (93/279)	18.15% (106/584)
Ampicillin	789 (83.84)	52.85 (417)	50.56% (180/356)	54.73% (237/433)
Amoxicillin–clavulanate	848 (90.21)	22.99 (195)	20.51% (73/356)	24.80% (122/492)
Piperacillin–tazobactam	940 (100)	15.85 (149)	14.89% (53/356)	16.44% (96/584)
Cefazolin/cephalotin	781 (83.08)	30.73 (240)	21.38% (62/290)	36.25% (178/491)
Ceftiofur	827 (87.98)	25.51 (211)	27.24% (79/290)	24.58% (132/537)
Tetracycline	889 (94.57)	46.68 (415)	62.08% (221/356)	36.40% (194/533)
Erythromycin	290 (30.85)	58.96 (171)		
Clindamycin	290 (30.85)	59.65 (173)		
Enrofloxacin	940 (100)	48.19 (453)	62.08% (221/356)	39.73% (232/584)
Trimethoprim–sulfamethoxazole	895 (95.21)	27.04 (242)	26.40% (94/356)	27.46% (148/539)

*Note*: in the first column, the number of isolates tested (excluding intrinsic resistances) and its percentage in relation with the total number of isolates is reported. In the second column, the percentage and the number of nonsusceptible isolates for every tested drug is described. In the third and fourth column, the nonsusceptibility percentages and number (number of nonsusceptible isolates/number of total isolates tested) of isolates identified as Gram positive and Gram negative is reported.

**Table 4:** Univariable logistic regression results from a study associating risk factors to antimicrobial resistance (AMR) or multidrug resistance (MDR) in 940 analyzed isolates collected from dogs and cats referred to a Veterinary University Hospital between November 2020 and September 2022.

Variables	AMR		MDR	
	*p* Value	OR (95% CI)	*p* Value	OR (95(%) CI)
Species (dog)	0.01^*∗*^	1.62(1.13–2.32)	0.66	1.07 (0.78–1.48)
Age (years)	0.52	0.99 (0.95–1.03)	0.36	0.99 (0.96–1.02)
Previous hospitalization/surgery in the past 30 days (yes)	<0.01^*∗*^	3.14 (1.83–5.39)	<0.01^*∗*^	2.76 (1.97–3.87)
Hospitalization at the time of the sampling (yes)	0.80	1.04 (0.75–1.45)	0.04^*∗*^	1.33 (1.02–1.73)
Hospitalization in intensive care unit (yes)	0.85	0.96 (0.65–1.42)	0.02^*∗*^	1.46 (1.06–2.00)
Surgery at the time of the sampling (yes)	0.70	0.93 (0.64–1.35)	0.67	0.94 (0.69–1.27)
Days of hospitalization at the time of the sampling (number of days)	0.10	1.06 (0.98–1.14)	<0.01^*∗*^	1.08 (1.03–1.14)
Invasive device use in the previous 90 days (yes)	<0.01^*∗*^	3.23 (2.07–5.05)	<0.01^*∗*^	2.62 (1.96–3.50)
Antimicrobial use in the past 90 days (yes)	<0.01^*∗*^	4.52 (3.07–6.65)	<0.01^*∗*^	2.892 (2.29–3.66)
Current antimicrobial use (yes)	0.18	1.23 (0.91–1.67)	<0.01^*∗*^	1.63 (1.28–2.07)

*Note*:  ^*∗*^Included in the multivariable analysis. Results considered significant (*p* < 0.05, marked with  ^*∗*^) were included in the multivariable analysis model. Odds ratios (OR) are reported with 95% confidence interval (CI).

**Table 5:** Results of the multivariable analysis results from a study to assess the association of risk factors included in the model to antimicrobial resistance (AMR) or multidrug resistance (MDR) in 940 analyzed isolates collected from dogs and cats referred to a Veterinary University Hospital between November 2020 and September 2022.

Variables	AMR		MDR	
	OR (95% CI)	*p* Value	OR (95% CI)	*p* Value
Species (dog)	1.55 (1.06–2.29)	0.02^*∗*^		
Previous hospitalization/surgery in the past 30 days (yes)				
Invasive device use in the previous 90 days (yes)	2.06 (1.29–3.30)	<0.01^*∗*^	1.76 (1.28–2.41)	<0.01^*∗*^
Antimicrobial use in the past 90 days (yes)	3.741 (2.516–5.564)	<0.01^*∗*^	2.47 (1.93–3.15)	<0.01^*∗*^
Current antimicrobial use (yes)			1.39 (1.08–1.79)	0.01^*∗*^

^*∗*^Significant according to the *p* value (<0.05). Results were considered significant with a *p* < 0.05. Odds ratios (OR) are reported with 95% confidence interval (CI). Only results with a significant *p* value (marked with  ^*∗*^) are shown.

**Table 6:** Nonsusceptibility percentages for every single tested drug considering only the 169 isolates identified as bacterial species belonging to the ESKAPE group, isolated from cats and dogs referred to a University Veterinary Hospital between September 2020 and November 2022.

	*E. faecium*	*S. aureus*	*K. pneumoniae*	*A. baumannii*	*P. aeruginosa*	*Enterobacter spp.*
Amikacin	Nt	6.67% (1/15)	8.51% (4/47)	0% (0/2)	11.11% (5/45)	9.52% (4/42)
Gentamicin	33.33% (6/18)	20.00% (3/15)	27.66% (13/47)	50.00% (1/2)	17.78% (8/45)	16.67% (7/42)
Ampicillin	83.33% (15/18)	86.67% (13/15)	Nt	Nt	Nt	Nt
Amoxicillin–clavulanate	83.33% (15/18)	26.67% (4/15)	48.94% (23/47)	Nt	Nt	Nt
Piperacillin–tazobactam	77.78% (14/18)	20.00% (3/15)	48.94% (23/47)	50.00% (1/2)	4.44% (2/45)	42.86% (18/42)
Cefazolin/cephalotin	Nt	33.33% (5/15)	65.96% (31/47)	Nt	Nt	Nt
Ceftiofur	Nt	40.00% (6/15)	61.70% (29/47)	Nt	Nt	47.62% (20/42)
Tetracycline	88.89% (16/18)	46.67% (7/15)	51.06% (24/47)	100.00% (1/1)	93.33% (42/45)	30.95% (13/42)
Erythromycin	Nt	33.33% (5/15)	Nt	Nt	Nt	Nt
Clindamycin	Nt	13.33% (2/15)	Nt	Nt	Nt	Nt
Enrofloxacin	100.00% (18/18)	46.67% (7/15)	70.21% (33/47)	50.00% (1/2)	86.67% (39/45)	59.52% (25/42)
Trimethoprim–sulfamethoxazole	44.44% (8/18)	6.67% (1/15)	53.19% (25/47)	100.00% (1/1)	Nt	47.62% (20/42)

*Note*: Nt stands for “non tested” due to intrinsic resistance. For each bacterial species, the total number of nonsusceptible isolates out of the total number of total isolates is reported in brackets.

**Table 7:** Nonsusceptibility percentages for every single tested drug considering the 138 isolates from specific specimens (blood cultures, urines from suspected HA UTIs, SSIs, and surgical implants), collected from cats and dogs referred to a University Veterinary Hospital between September 2020 and November 2022.

	Blood cultures (n = 49)	Urines from suspected HA UTIs (N = 42)	SSIs and surgical implants (n = 49)
Amikacin	11.36% (5/44)	12.12% (4/33)	12.50% (5/40)
Gentamicin	26.67% (12/45)	41.02% (16/39)	45.45% (20/44)
Ampicillin	58.82% (20/34)	75.86% (22/29)	70.27% (26/37)
Amoxicillin–clavulanate	35.13% (13/37)	45.95% (17/37)	45.95% (17/37)
Piperacillin–tazobactam	24.49% (12/49)	33.33% (14/42)	34.69% (17/49)
Cefazolin/cephalotin	51.35% (19/37)	61.29% (19/31)	57.58% (19/33)
Ceftiofur	46.67% (21/45)	51.51% (17/33)	59.52% (25/42)
Tetracycline	67.39% (31/46)	69.05% (29/42)	67.35% (33/49)
Erythromycin	73.33% (11/15)	81.82% (9/11)	68.42% (13/19)
Clindamycin	73.33% (11/15)	81.82% (9/11)	68.42% (13/19)
Enrofloxacin	55.10% (27/49)	71.43% (30/42)	73.47% (36/49)
Trimethoprim–sulfamethoxazole	36.95% (17/46)	51.28% (20/39)	56.52% (26/46)
% AMR	81.63% (40/49)	90.48% (38/42)	93.88% (46/49)
% MDR	61.22% (30/49)	69.05% (29/42)	73.47% (36/49)

*Note*: the total number of nonsusceptible isolates out of the total number of tested isolates is reported in bracket.

## Data Availability

The data that support the findings of this study are available from the corresponding author upon request.

## Supplementary Materials

Supplementary Materials Table S1: summarizes the standard procedures of bacteriological culture used in the study. Table S2: lists the antimicrobials tested for the antimicrobial susceptibility testing.
