# Supplementary material for: Monitoring the Prevalence of Antimicrobial Resistance in Companion Animals: Results from Clinical Isolates in an Italian University Veterinary Hospital
Source: Transbound Emerg Dis. 2023 Sep 14;2023:6695493. doi: 10.1155/2023/6695493 (PMC12016709; doi:10.1155/2023/6695493)
Supplement: Supplementary Materials — Table S1: summarizes the standard procedures of bacteriological culture used in the study. Table S2: lists the antimicrobials tested for the antimicrobial susceptibility testing. [file 6695493.f1.docx]

**Table S1. List of culture media, condition and temperature used depending on the specimen type.**

| **Specimen type** | **Media** ^a^ | **Incubation** |
| --- | --- | --- |
| Urine ^b^; ear swab | Blood Agar, Cled, Mac Conkey | Aerobic conditions |
| vaginal/uterine swab; wound; respiratory tract lavage; surgical site infection | Blood Agar, Cled, Mac Conkey | Aerobic conditions |
|  | Columbia Agar | Capnophilic conditions |
|  | Columbia Agar | Anaerobic conditions |
| bile; biopsy; blood culture ^c^; abdominal/pleural/peritoneal effusion, exudate ^d^, abscess. | Blood Agar, Cled, Mac Conkey | Aerobic conditions |
|  | Columbia Agar | Capnophilic conditions |
|  | Columbia Agar, Wilkins-Chalgren Agar | Anaerobic conditions |

**FOOTNOTE:**

^a^ All the culture media were purchased from Oxoid, Germany, and prepared following manufacturer's instructions. Blood Agar and Columbia were made with 5% horse blood.

^b^ Obtained both by cystocentesis and catheterization.

^c^ Only for blood cultures found to be positive after an initial incubation at 37 °C for 7 days in a blood culture bottle (Signal Blood Culture System; Oxoid, Milan, Italy), following manifacturer's instructions.

^d^ Abcess/exudate were defined by the submitting veterinarian based on macroscopic aspect

**TABLE S2. List of tested antimicrobials divided for antimicrobial class.**

| Aminoglycosides | Amikacin 30 μg |
| --- | --- |
|  | Gentamicin 10 μg (120 μg for *Enterococcus* spp. isolates) |
| Penicillins +/- beta-lactamases inhibitors | Ampicillin 10 μg |
|  | Amoxicillin- clavulanate 30 μg |
|  | Piperacillin-tazobactam 110 μg |
| Cephalosporins | Cefazolin/cephalothin 30 μg |
|  | Ceftiofur 30 μg |
| Tetracyclines | Tetracycline 30 μg |
| Macrolides | Erythromycin 15 μg |
| Lincosamides | Clindamycin 2 μg |
| Fluoroquinolones | Enrofloxacin 5 μg |
| Sulfonamides + dihydrofolate reductase inhibitors | Trimethoprim- sulfamethoxazole 1.25/23.7 μg |
